# GROUP RESIDENTIAL TRAINING OF BLIND OLDER ADULTS OFFERED BY VISUALLY IMPAIRED AND SIGHTED PROFESSIONAL TRAINERS

**DOI:** 10.1093/geroni/igad104.3376

**Published:** 2023-12-21

**Authors:** Nancy Miller, Frankie Ann Marcille, Sierra Storm, Cathleen McGuire

**Affiliations:** VISIONS/Services for the Blind and Visually Impaired, New York City, New York, United States; VISIONS/Services for the Blind and Visually Impaired, New York City, New York, United States; VISIONS/Services for the Blind and Visually Impaired, New York City, New York, United States; VISIONS/Services for the Blind and Visually Impaired, New York City, New York, United States

## Abstract

VISIONS/Services for the Blind and Visually Impaired piloted a 6-day residential group training for 6 legally blind older adults ages 57-80, self identified as Caucasian or African-American. Assessments occurred in five core areas: personal, home, financial and meal management and communication skills. Mobility and family care skills were also assessed. One legally blind and one sighted professional instructor offered the training along with adaptive equipment and training on how to use it. Each participant received follow up visits in their home to measure how well they retained the skills taught and how well they were safely using them. Referrals were made to VISIONS occupational therapists to address other chronic health conditions, social workers for adjustment to blindness counseling or low vision optometrists for additional follow up. Offering intensive residential group training with peers resulted in documented improvement in ADL/IADL skills and increased social connections. Group training, even when participants had varied skill levels, education, age of onset of vision loss, and eye diseases, proved successful. One participant framed his completion certificate and hung it in his living room. The value of learning new skills with a peer group was confirmed as well as the willingness for older adults with legal blindness to attend a 6-day residential program. Providing group training helps to address the long waitlist for services with a national personnel shortage of nationally certified vision rehabilitation therapists to serve the growing older blind. population. Group training also effectively utilizes the limited government funding for training older blind adults.

